# Antioxidant and Antimicrobial Activities of 7-Hydroxy-calamenene-Rich Essential Oilsfrom *Croton cajucara* Benth

**DOI:** 10.3390/molecules18011128

**Published:** 2013-01-16

**Authors:** Mariana M. B. Azevedo, Francisco C. M. Chaves, Catia A. Almeida, Humberto R. Bizzo, Rafael S. Duarte, Galba M. Campos-Takaki, Celuta S. Alviano, Daniela S. Alviano

**Affiliations:** 1 Chemistry Institute, Federal University of Rio de Janeiro (UFRJ), CT, Ilha do Fundão, Rio de Janeiro 21941-909, RJ, Brazil; 2 EMBRAPA Western Amazon, Rodovia AM 10, km 29, Manaus 69010-970, AM, Brazil; 3 Institute of Microbiology Paulo de Góes, Federal University of Rio de Janeiro (IMPG-UFRJ), CCS, Ilha do Fundão, Rio de Janeiro, RJ 21941-590, Brazil; 4 EMBRAPA Food Agroindustry, Avenida das Américas 29501, Rio de Janeiro 23020-470, RJ, Brazil; 5 Center for Research in Environmental Sciences, Catholic University of Pernambuco, Rua do Príncipe 526, Recife 50050-900, PE, Brazil

**Keywords:** *Croton cajucara*, 7-hydroxycalamenene, essential oils, antimicrobial activity, antioxidant activity

## Abstract

*Croton cajucara* is a shrub native to the Amazon region locally known as “sacaca”. Two morphotypes are known: white and red “sacaca”. The essential oils (EO) obtained by hydrodistillation from leaves of the red morphotype were, in general, rich in 7-hydroxycalamenene (28.4%–37.5%). The effectiveness of these EO regarding the antimicrobial activity against pathogenic microorganisms was initially investigated by the drop test method, showing significant inhibition zones. Among the microorganisms tested, the essential oils rich in 7-hydroxycalamenene were more effective against methicillin resistant *Staphylococcus aureus* (MRSA), *Enterococcus faecalis*, *Mycobacterium tuberculosis*, *M. smegmatis*, *Mucor circinelloides* and *Rhizopus oryzae*. The minimum inhibitory concentrations (MIC) of the oils were determined using the broth dilution assay. It was possible to observe that 7-hydroxycalamenene-rich oils presented high antimicrobial activity, with MIC of 4.76 × 10^−3^ μg/mL for MRSA, 4.88 μg/mL for *M. tuberculosis*, 39.06 μg/mL for *M. smegmatis*, and 0.152 μg/mL for *R. oryzae* and 3.63 × 10^−8^ μg/mL for *M. circinelloides*. The antioxidant activity of this EO suggests that 7-hydroxycalamenene provides more antioxidant activity according with EC_50_ less than 63.59 μg/mL. Considering the bioactive potential of EOs and 7-hydroxycalamenene could be of great interest for development of antimicrobials for therapeutic use in treatment of bacterial and fungal infections in humans and/or veterinary practice.

## 1. Introduction

*Croton cajucara* Benth. (Euphorbiaceae) has been a very important traditional medicinal plant in Brazil. It occurs widely in the Brazilian Amazon rainforest region where it is popularly known as “sacaca”. Infusions of the stem bark have been used for the treatment of liver and kidney disorders, diabetes, diarrhea, stomach ache, fever, jaundice, hepatitis, malaria and also to lower blood cholesterol. Although the use of leaves for losing weight has been encouraged in the Brazilian Amazon region, toxic hepatitis frequently appears as a side effect. This observation may be correlated to the chronic use required for losing weight, since a lack of acute hepatotoxicity has been described and this toxicological effect was not noticed [1,2].

Two morphotypes of *C. cajucara* are known: white “sacaca” and red “sacaca”, mainly identified by young leaf colour and steams [3]. In general, essential oils from the white morphotype are rich in linalool, while those from red morphotype are rich in 7-hydroxycalamenene, although some exceptions have been observed and, therefore, this substance cannot be used as a chemical marker [3,4]. The linalool-rich essential oil from the leaves of *C. cajucara* has been shown to be very toxic for *Leishmania amazonensis* and *Candida albicans* [5,6].

The objective of this work was to evaluate the antimicrobial and antioxidant activities of the essential oils from the leaves of *Croton cajucara* with different contents of 7-hydroxycalamenene.

## 2. Results and Discussion

The average oil yield obtained was 0.65% (dried basis). The major compounds present in the essential oils from *C. cajucara* used are shown in Table 1. Quantitative and/or qualitative variations were observed among samples of red “sacaca”. For one individual, no 7-hydroxycalamenene was detected and α-pinene was the major component of the oil, as shown in Table 1.

The results for antimicrobial activity of the *C. cajucara* essential oils are presented in Table 2. For filamentous fungi the largest inhibition zone was obtained with 7-hydroxycalamenene-rich essential oils. For *M. smegmatis*, the best results were with SV002 (without 7-hydroxycalamenene and with α-pinene), and SV004 and SV005 (over 30% of 7-hydroxycalamenene). Although, the lowest inhibition zone was obtained through SV001 (containing 7-hydroxycalamenene and α-pinene). For *E. faecalis*, *S. epidermidis*, MRSA and *L. casei* the largest inhibition zones were all associated with a high content of 7-hydroxycalamenene, which may indicate that this substance might be responsible for the inhibitory activity. The difference of inhibition zones can be justified by differences of boiling point (bp) values of compounds (e.g., bp of α-pinene 52.5°). It is important to observe those values because the compounds present in the essential oil can change the atmosphere and interfere on microbial growth. Tullio *et al.* (2006) [7] showed that *Mucor* spp. and *Rhizopus* spp. exhibited susceptibility to pine essential oil (55.7% of α-pinene) vapor.

**Table 1:** Main components from *C. cajucara* essential oils.

Components	Samples (in%)				
	SV001	SV002	SV003	SV004	SV005
a-Pinene	7.5	24.7	0.1	0.5	t
Linalool	6.3	11.6	11.0	9.9	13.2
7-Hydroxycalamenene	37.5	n.d.	28.4	30.9	32.9
b-Caryophyllene	4.1	5.7	2.8	4.0	2.6

n.d.: not detected; t: trace (less than 0.1%).

**Table 2:** Inhibition zones (in mm) for *C. cajucara* essential oils.

Microorganisms	SV001	SV002	SV003	SV004	SV005
*A. fumigatus*	4.8	3.2	4.5	4.6	5
*A. niger*	5.7	3.4	5.6	5.9	6
*A. ochraceus*	4.5	3.1	4.5	4.7	5
*F. solani*	3.1	2.2	3.4	3.5	3.5
*M. gypseum*	14	3	13	14	14
*M. circinelloides*	8	15	6	10	10
*R. oryzae*	6	12	5	8	10
*T. rubrum*	5.8	0	5.5	5.7	6
*M. smegmatis*	10	24	12	18	18
*E. faecalis*	10	6	8	6	9
*S. epidermidis*	6	9	7	8	20
*S. aureus* MRSA	7	5	7	13	38
*L. casei*	8	7	35	11	14
*C. albicans*	6	10	7	8	8

For TLC and, therefore, bioautography, linalool and 7-hydroxycalamenene presented quite close R_f_ values (0.43 and 0.58, respectively), so the oil SV002 was chosen for bioautography to evaluate only the effect of linalool on the microorganisms tested. For this sample (with 11.6% of linalool) inhibition was observed for *C. albicans* but not for MRSA. While for SV001 showed strong inhibition in both microorganisms.

In Table 3 the MIC values for *M. smegmatis*, *M. tuberculosis*, *S. aureus* MRSA, *C. albicans*, *M. circinelloides* and *R. oryzae* can be observed. The microorganisms tested were susceptible at different concentrations to the oils rich in 7-hydroxycalamenene. This result may be due to differences in composition and, consequently, the interaction of the essential oil with the culture medium. The activity of 7-hydroxycalamenene-rich essential oil is confirmed by the values of MIC of 7-hydroxycalamenene isolated.

**Table 3:** MICs values (in μg/mL) of essential oil samples.

Microorganisms	SV001	SV002	SV003	SV004	SV005	7-OH
*M. smegmatis*	39.06	5000	78.12	156.25	156.25	39.06
*M. tuberculosis*	4.88	4.88	4.88	4.88	4.88	312.5
MRSA	0.019	na	0.019	0.004	0.001	39.06
*C. albicans*	1.22	1250	156.25	0.001	0.038	78.125
*M. circinelloides*	nd	nd	nd	nd	3.63 × 10^−8^	19.53
*R. oryzae*	nd	nd	nd	nd	0.152	39.06

na: no activity; nd: not determined; 7-OH: 7-hydroxycalamenene.

According to Sun *et al.* (2002) [8] the MICs_80_ of posaconazole, amphotericin B and itraconazole were 0.125, 0.25 and 0.25 μg/mL for *Mucor ramosissimus* and 8, 0.25 and 8 μg/mL for *Mucor circinelloides*, respectively. Comparing the above with the MICs obtained with the essential oils of five samples of red “sacaca”, the latter showed more effectivity. The MICs of amphotericin B, itraconazole and posaconazole for *R. oryzae* were 1, 0.5 and 0.25 μg/mL, respectively [9]. These results when compared to the MIC obtained with sample 5 from red “sacaca” show that this is more effective than amphotericin B, itraconazole and posaconazole.

*C. albicans* was susceptible to the oils tested, achieving very close results when compared to a MIC of 1.6 μg/mL for amphotericin B. For MRSA the 7-hydroxycalamenene-rich oils were far more active than reference drugs, with MICs ranging from 1.19 to 19.07 ng/mL, against 250 ng/mL for ciprofloxacin [10].

The MICs for *M. tuberculosis* and *M. smegmatis* have shown promising values when compared with Stephan *et al.* (2004) [11], whose MICs of various antibiotics for *M. smegmatis* were less effective than the red “sacaca” essential oils. According to data provided by the authors the essential oils are more effective or as effective as isoniazid, cycloserine, erythromycin and the cephalosporins. Only sample SV002 (without 7-hydroxycalamenene) had weak activity against *M. smegmatis*. According Wiid *et al.* (1999) [12], Maus *et al.* (2005) [13] and Shandil *et al.* (2007) [14] the major antimicrobial MICs for *M. tuberculosis* are 0.5 μg/mL for moxifloxacin, 0.5 μg/mL for ofloxacin, 0.1 μg/mL for sparfloxacin, 0.1 μg/mL for ciprofloxacin, 10 μg/mL for capreomycin, 10 μg/mL for viomycin, 5 μg/mL for kanamycin, 4 μg/mL for amikacin and 0.05 μg/mL to isoniazid. Despite the MICs of some antimicrobial drugs being more effective, the results showed the activity of essential oils was as effective or more effective than amikacin, kanamycin, capreomycin and viomycin. With these results, they can be considered effective since substances with antagonistic activity may exist in the essential oils. 

The microbicidal activity of 7-hydroxycalamenene at MIC values was observed for all microorganisms tested. Although the essential oil results are very promising, 7-hydroxycalamenene shows quite increased MIC values. Although the high activity of essential oil could be justified, as due to sensitivity of the differences between the MICs of essential oils and 7-hydroxycalamenene, our results demonstrate that 7-hydroxycalamenene is active on a broad spectrum of microorganisms.

It is usually considered that strong activity corresponds to MIC values between 0.05 and 0.50 mg/mL, moderate activity values between 0.6 and 1.50 mg/mL and above 1.50 mg/mL as weak activity [15]. According to this classification it could be stated that oils from the red morphotypes with high content of 7-hydroxycalamenene and 7-hydroxycalamenene isolated present high activity against the most of microorganisms tested. 

The antioxidant activity was evaluated after TLC of the oil. It was possible to identify regions containing substances with activity even after 45 min of application of DPPH. The antioxidant activity was associated to 7-hydroxycalamenene (center spot) and β-caryophyllene (upper spot).

**Figure 1:**
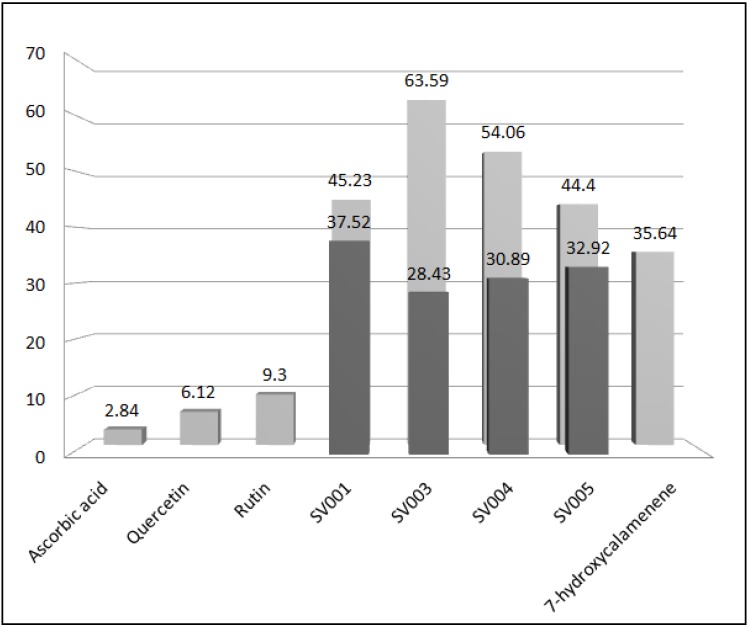
Comparison between presence of 7-hydroxycalamenene (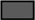) and EC_50_ (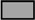) of standards, essential oils and 7-hydroxycalamenene.

A quantitative evaluation was performed (Figure 1), and the logarithmic and linear regressions were obtained for the calculation of EC_50_. These values were: SV001—45.23 μg/mL, SV003—63.59 μg/mL, SV004—54.06 μg/mL, SV005—44.4 μg/mL and 7-hydroxycalamenene—35.64 μg/mL. The EC_50_ of ascorbic acid, quercetin and rutin were, respectively, 2.84 μg/mL, 6.12 μg/mL and 9.3 μg/mL.

According to these results, we can suggest that in all samples with 7-hydroxycalamenene tested present promising antioxidant activity.

## 3. Experimental

### 3.1. Plant Material

Five individuals of red morphotype of *C. cajucara* were kept in the germplasm bank under the same cultivation practices. The leaves were collected between 8 h and 9 h. A voucher specimen was deposited at EMBRAPA Amazônia Ocidental Herbarium (registry IAN 165013).

### 3.2. Chemicals

All solvents used were spectroscopic grade from Tedia (Fairfield, OH, USA). Reagents were from Sigma-Aldrich (St. Louis, MO, USA). Column and planar chromatographic products were obtained from Merck (Darmstadt, Germany).

### 3.3. Extraction and Analysis of the Essential Oils

Extraction was performed by hydrodistillation in a modified Clevenger apparatus for 4 h, according Pereira *et al.* (2011) [4]. The oils were analyzed on an Agilent (Palo Alto, CA, USA) 6890N gas chromatograph fitted with a 5% phenyl—95% methylsilicone (HP-5, 30 m × 0.32 mm × 0.25 μm) fused silica capillary column. The oven temperature was programmed from 60 °C to 240 °C (3 °C/min), and hydrogen was used as carrier gas (1.4 mL/min). It was injected 1.0 μL of a 1% solution of the oil in dichloromethane, in split mode (1:100). Injector was kept at 250 °C and detector (FID) at 280 °C. All analyses were performed in triplicate.

Mass spectra were obtained in an Agilent 5973N system operating in electronic ionization mode (EI) at 70 eV, with scan mass range of 40–500 *m/z*. Sampling rate was 3.15 scan/s. Ion source was kept at 230 °C, mass analyzer at 150 °C and transfer line at 260 °C. The mass detector was coupled to an Agilent 6890 gas chromatograph fitted with a low bleeding 5% phenyl–95% methylsilicone (HP-5 MS, 30 m × 0.25 mm × 0.25 μm) fused silica capillary column. Injection procedure and oven temperature program were the same as above. Helium was the carrier gas, at 1.0 mL/min. 

Linear retention indices (LRI) were measured by injection of a series of *n*-alkanes (C_7_-C_26_) in the same column and conditions as above for GC analyses [16]. Identification of the oil components was based on computer search using the Wiley 6th ed. Library of Mass Spectral Data and by comparison of their calculated LRI with literature data [17]. 7-Hydroxycalamenene was isolated according to Azevedo *et al.* (2012) [10]. Linalool was also identified by injection of an authentic standard.

### 3.4. Microorganisms

The strains used to examine the antimicrobial activity of essential oils were methicillin-resistant *Staphylococcus aureus* (MRSA-BMB9393); *Enterococcus faecalis*, *Staphylococcus epidermidis* and *Lactobacillus casei* (Hospital Clementino Fraga Filho, UFRJ-HUCFF/UFRJ); *Mycobacterium smegmatis* (00061) and *Mycobacterium tuberculosis* H37Rv (ATCC 27294); *Candida albicans* (ATCC 24433); *Aspergillus fumigatus* (ATCC 16913), *Aspergillus niger* (HUCFF/UFRJ), *Aspergillus ochraceus* (ATCC 22947), *Mucor circinelloides* (LIKA0066), *Rhizopus oryzae* (UCP1506), *Trichophyton rubrum* (T544), *Fusarium solani*, *Microsporum gypseum* (HUCFF/UFRJ). The microorganisms were stored in specific culture media slanted tubes at 4 °C. Prior to use, the microorganisms were grown in BHI agar for 24 h (for bacteria and yeast), in Potato Dextrose agar (PDA) for 5 days (for filamentous fungi); in Mueller-Hinton agar for 7 days (for *M. smegmatis*) and in Middlebrook 7H10 agar for 28 days (for *M. tuberculosis*).

### 3.5. Antimicrobial Analysis of the Essential Oils

The antimicrobial assay was carried out by the drop agar diffusion method. The microorganisms were spread over Petri plates containing BHI agar, for bacteria and *Candida*, 7H10, for mycobacterium, and PDA for filamentous fungi. After 10 min, 10 μL of the essential oils was added to the center of each plate. All plates were incubated according to the microorganisms, after which the diameter of inhibition zone (in mm) was measured [18].

### 3.6. Thin-layer Chromatography (TLC) and Bioautography

All the oils were applied on Merck (Darmstadt) silica-gel G60 F_254_ chromatoplates and eluted with hexane-ethyl acetate (9:1). Spots were observed under UV light (254 nm), then stained with sulphuric anisaldehyde and heating. For the bioautography the TLC was repeated, but after elution and solvent drying the plates were exposed to UV for 20 min, then added to sterile Petri plates and immediately covered with 6 mL of BHI previously inoculated with the microorganisms to be tested (*circa* 10^6^ to 10^8^ cells/mL). After incubation period, the plates were observed and the inhibition zones were marked with 3-(4,5-dimethyl-2-thiazolyl)-2,5-diphenyl-2H-tetrazolyum bromide (MTT) [19].

### 3.7. Minimal Inhibitory Concentration (MIC) and Minimum Microbicidal/Microbiostatic Concentration Assays

The *in vitro* susceptibility was determined by the minimum inhibitory concentration determination method. The MICs of 7-hydroxycalamenene-rich essential oils were determined by two-fold serial dilution as described by the Clinical and Laboratory Standards Institute (M27-A2, M38-A, M24-A, M7-A6) [20,21,22,23]. 

In the first well 0.5 μL of essential oil was applied individually to initiate the serial microdilution and microbial suspension equivalent to 0.5 McFarland standard. To confirm the results 30 μL of 0.005% resazurin in PBS (pH 7.2) were used. The negative and positive controls comprised pure growth media and inoculated growth media without test agent, respectively. The experiments were performed in triplicate and at least three times.

The microbicidal/microbiostatic concentrations were determined by sub-culturing the test dilutions onto a specific fresh solid media and incubating further for 18–72 h, for bacteria and fungi, and 7–28 days for mycobacteria. The highest dilution that yielded no bacterial/fungal growth on solid medium was taken as microbicidal/microbiostatic concentrations [24].

### 3.8. Antioxidant Activity

The antioxidant activity was evaluated qualitatively [25] by application of 0.5 μL of each essential oil and 7-hydroxycalamenene on a plate of silica-gel 60 F_254_ and eluting as described above. The plates were treated with a 0.2% methanolic solution of DPPH and the read just after spraying and after 45 min. For quantitative evaluation, serial dilutions in ethanol of each essential oil were performed [26]. Reading of antioxidant activity was obtained after addition of 60 μL of ethanolic solution of 0.3 mM DPPH and under 492 nm wavelength after 30 min. Ascorbic acid, rutin and quercetin were used as reference standards. The result was converted to percentage antioxidant activity using the following formula:

AA% = 100 − {[(Abs_sample_ − Abs_Blank_) × 100]/(Abs_Control_ − Abs_Blank_)}

The EC_50_ values, half of the antioxidant activity, were calculated from logarithmic or linear regression, where the abscissa represents the concentration of essential oil tested and ordered the average percentage of antioxidant activity from five separate tests.

## 4. Conclusions

Samples of *C. cajucara* essential oils presented differences in the chemical composition, although all samples are rich in 7-hydroxycalamenene (except SV002). The oils containing high amounts of this compound were effective against methicillin-resistant *S. aureus*, *M. smegmatis*, *M. tuberculosis*, *M. circinelloides* and *R. oryzae*. In addition, it presented as a potent antioxidant.

The assessment of antibacterial, antimycobacterial and antifungal activities showed that the most active compound was 7-hydroxycalamenene. Nevertheless, it’s necessary to confirm its viability in the formulation of new drugs by toxicity assays of 7-hydroxycalamenene as well as complementary studies aiming to investigate the possible mechanisms of action and the activity of this substance for other clinically important microorganisms.
